# Editorial: Neuroanatomy of cognition

**DOI:** 10.3389/fnana.2024.1376610

**Published:** 2024-03-08

**Authors:** Mariana Bendersky, Lucia Alba-Ferrara

**Affiliations:** ^1^University of Buenos Aires, Buenos Aires, Argentina; ^2^Estudios en Neurociencias y Sistemas Complejos (CONICET), Florencio Varela, Argentina; ^3^Austral University, Buenos Aires, Argentina

**Keywords:** neuroanatomy, cognition, language, consciousness, sleep, neuroscience

Neuroanatomy of cognition is a complex and evolving field of study that explores the structural and functional bases of cognitive processes. Research in this area delves into the neural structures and pathways involved in cognitive domains, such as attention, memory, language, and decision-making. Studying the neuroanatomical underpinnings of cognition is crucial to understand normal brain function and addressing neurological disorders affecting cognition. This knowledge can help to develop more effective treatments and interventions for various cognitive conditions.

Furthermore, neuroscience research has demonstrated the powerful role of experience in shaping the mind, brain, and body, which can help detect abnormalities before they become evident.

Research on neuroanatomy of cognition utilizes various approaches and techniques to study the neural structures and pathways involved in cognitive processes. In this Research Topic, a diverse range of methodologies, such as fMRI, lesion network mapping, MRI tractography, genetic strategies, and viral-based directional tracers, are employed to reveal the neuroanatomy of cognition and its implications for brain disorders. The role of subcortical structures in cognition, an area of active research that has made significant advancements in recent years, is highlighted. The interaction between subcortical and cortical structures in cognitive processing occurs through complex and interconnected networks. The included articles demonstrate that cortico-subcortical interaction influences not only motor behavior and consciousness but also cognition and emotion.

Janko et al. reviewed the localization of finger representations as documented in various human fMRI studies. Primarily situated in Brodmann areas 3b (BA3b), 2 (BA2), and 1 (BA1), these maps exhibited a consistent pattern of organization from lateral to medial and inferior to posterior within the cortex, corresponding to the arrangement of digits from D1 to D5. The findings revealed varying degrees of spatial overlap between the representations of different fingers within the brain. The thumb exhibited the smallest receptive field and the greatest cortical magnification. These findings enhance our comprehension of the neural mechanisms that underlie finger dexterity and the control of fine motor skills, having pivotal implications for the development of rehabilitation and training programs aimed at improving and optimizing these abilities.

While central sleep apnea (CSA) has traditionally been associated with brainstem centers, Yuan et al. discovered that supratentorial injuries can also lead to this condition. The authors pinpointed the neuroanatomical foundations that regulate breathing during sleep and whose damage underlies the pathophysiology of CSA, employing a new technique called lesion network mapping. They observed that lesions causing CSA demonstrated positive connectivity with the cingulate gyrus, bilateral cerebellum, and bilateral thalamus, along with negative connectivity with the bilateral middle occipital gyrus. These findings have the potential to improve diagnosis, clinical management, neuroanatomical targets and outcomes for individuals affected by this sleep disorder. Besides, the application of lesion network mapping offers a promising approach for investigating other neurological syndromes.

Ham and Augustine used genetic strategies and viral-based directional tracers to examine the spatial distribution and connectivity patterns of claustrum (CLA) neuron populations that project to the retrosplenial cortex, primary motor cortex, or basolateral amygdala. The study revealed that each neuron populations occupy distinct spatial locations within the CLA complex and send widespread projections to multiple and specific targets. So, the CLA may play a role in orchestrating communication between distributed brain networks and integrating sensory, motor, and cognitive information. This integrative function could contribute to higher-order cognitive processes such as attention, perception, and decision-making.

According to the mesocircuit hypothesis, the internal globus pallidus (GPi) has been implicated in prolonged disorders of consciousness (DOC) due to its excessive inhibition of central thalamic nuclei. Zheng and Monti propose that the external globus pallidus (GPe), involved in modulating arousal, sleep, and GABA dysfunction, should also be included in this model. They used data from the Human Connectome Project to investigate pallidal connections via probabilistic tractography, and found that the GPe have direct connections with the prefrontal cortex and central thalamic nuclei, while the GPi predominantly connects with sensorimotor subregions of the thalamus. These findings emphasize the need to include the GPe in mechanistic models of consciousness in brain injury.

In summary, this Research Topic included valuable insights into specific cognitive functions, such as the cortical control of finger movement, the supratentorial mechanisms that regulate breathing during sleep, the specific functions of subregions of the claustrum, and the plasticity of pragmatic language networks in epilepsy. These studies highlight the importance of individualized patterns of reorganization and offer promising approaches for future research. Ultimately, the field of neuroanatomy of cognition holds great potential for enhancing our understanding of the brain and improving the lives of individuals affected by neurological conditions.

## Author contributions

MB: Writing – original draft, Writing – review & editing. LA-F: Writing – original draft, Writing – review & editing.

